# Alzheimer's disease: 120 years of research and progress

**DOI:** 10.25122/jml-2022-0111

**Published:** 2023-02

**Authors:** Vlad Alexandru Ciurea, Razvan-Adrian Covache-Busuioc, Aurel George Mohan, Horia Petre Costin, Victor Voicu

**Affiliations:** 1 Neurosurgery Department, Carol Davila University of Medicine and Pharmacy, Bucharest, Romania; 2 Neurosurgery Department, Sanador Clinical Hospital, Bucharest, Romania; 3 Carol Davila University of Medicine and Pharmacy, Bucharest, Romania; 4 Department of Neurosurgery, Bihor County Emergency Clinical Hospital, Oradea, Romania; 5 Neurosurgery Department, Faculty of Medicine, Oradea University, Oradea, Romania; 6 Pharmacology, Toxicology and Clinical Psychopharmacology Department, Carol Davila University of Medicine and Pharmacy, Bucharest, Romania; 7 Romanian Academy, Bucharest, Romania

## THE HISTORY OF DEMENTIA: FROM ANTIQUITY TO MODERN TIMES

Cognitive disorders have fascinated people since antiquity, as they often present with memory impairment and diminished social interactions. Dementia was the common name for all cognitive disorders that had an impact on the psychological and social aspects of the patient long before Alzheimer's disease was discovered. The history of dementia can be traced back to the Greco-Roman period when it was considered a disease of the elders. The term 'dementia' is derived from the Latin word "demens", which describes a person being out of their mind [1]. The famous philosopher Pythagoras classified human life into five stages, with the last two age groups being regarded as "senium", involving both psychological and physical deterioration [2]. Meanwhile, Hippocrates supposed that mental decline was a consequence of decreased body fluids in the brain of elders, leading progressively to an unfavorable outcome when the patient cannot memorize or have any social interactions [2].

Until the 19^th^ century, there were only a few papers written about dementia in medical books and writings, and poets and playwrights described characters with cognitive disorders without explicitly stating dementia as the pathology involved. However, in the 19^th^ century, thanks to Philippe Pinel, a French physician regarded as the "Father of modern psychiatry", who militated against the prosecution of alienated patients, cognitive disorders were transformed into clinical pathologies that could be treated [3]. The 20^th^ century marked a significant moment in the classification of cognitive disorders as clinical pathologies. During this time, neurosyphilis was the main cause of dementia, but it was easily curable if diagnosed early.

## ALOIS ALZHEIMER: LIFE AND CONTRIBUTIONS TO PSYCHIATRY AND NEUROSCIENCE

Alzheimer's disease was discovered in the early 20^th^ century by the German psychiatrist and neuropathologist Alois Alzheimer after a study that started in 1901.

**Figure 1:**
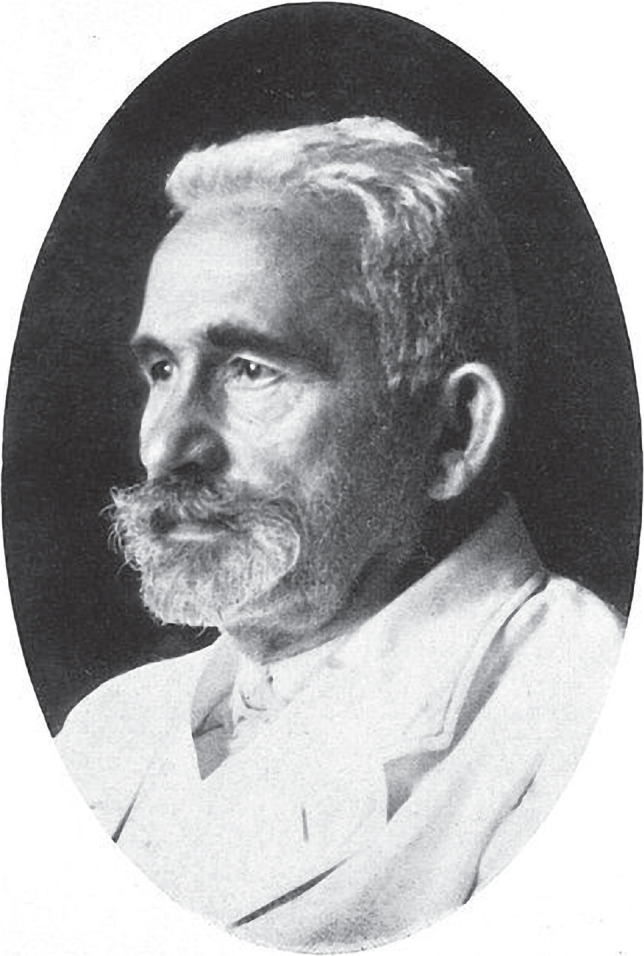
Emil Kraepelin, about 1920. © Munchener Medizinische Wochenschrift (1926).

Alois Alzheimer, born in 1864 in Marktbreit, Bavaria, studied medicine at the University of Berlin, the University of Tübingen, and the University of Würzburg, where he graduated in 1887 with a Doctor of Medicine degree. He later assumed a position at the Frankfurt asylum, where he had the opportunity to meet Emil Kraepelin (Figure 1), a well-known German psychiatrist who was investigating psychosis in senile patients at that time. Kraepelin became a mentor to Alzheimer and is regarded as the founder of modern scientific psychiatry, psychopharmacology, and psychiatric genetics, advocating for biological and genetic malfunction as the etiology of cognitive disorders [4].

In 1908, Alzheimer became a professor at Ludwig Maximilian University of Munich, and in 1912, he became a psychiatry professor and director of the neurologic and Psychiatric Institute in Breslau for the Silesian Friedrich Wilhelm University in Breslau. Unfortunately, despite his numerous academic achievements, Alzheimer's life was cut short when he died on December 19^th^, 1915, at the age of 51 due to a streptococcal infection that led to heart failure. Nevertheless, his contributions to the field of psychiatry and neuroscience continue to inspire researchers and clinicians to this day.

## THE FIRST STUDY ON ALZHEIMER’S DISEASE AND THE FIRST PATIENT DIAGNOSED

**Figure 2:**
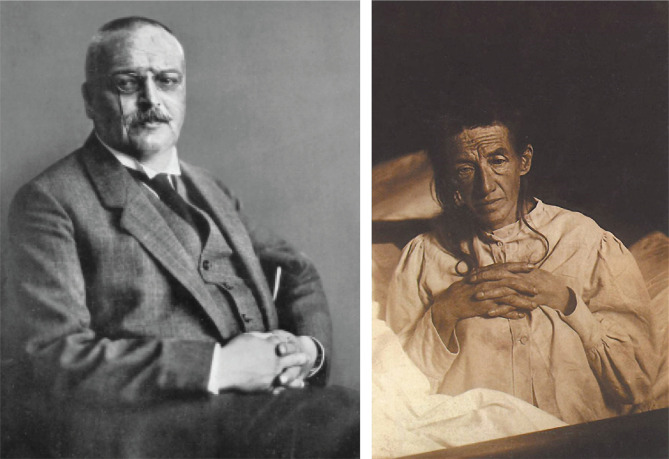
Alois Alzheimer, about 1909. © Archive for History of Psychiatry, Department of Psychiatry University of Munich (left) and Auguste Deter (right).

In 1901, Alois Alzheimer became interested in a patient at Frankfurt asylum named Auguste Deter (Figure 2), a 51-year-old female with unique symptomatology compared to normal dementia cases. She presented progressive confusion, sleep disorders, and, most importantly, memory loss [5]. Alzheimer became fascinated by this clinical case as the patient was not so old, and she did not present arteriosclerosis since no signs of vessel structure were found. Moreover, her symptoms developed progressively, which excluded the case of an arteriosclerotic brain [6].

Alzheimer described Auguste Deter's symptomatology in a paper for the "37^th^ Meeting of South-West German Psychiatrists in Tubingen", but unfortunately, it was not appreciated by the academic community. However, it was later published in 1907 entitled "Über eigenartige Krankheitsfälle des späteren Alters" (*On certain peculiar diseases of old age*).

He stated the following report: "*Her memory is seriously impaired. If objects are shown to her, she names them correctly, but almost immediately afterwards she has forgotten everything. When reading a test, she skips from line to line or reads by spelling the words individually, or by making them meaningless through her pronunciation. In writing, she repeats separate syllables many times, omits others and quickly breaks down completely. In speaking, she uses gap-fills and a few paraphrased expressions ("milk-pourer" instead of cup); sometimes it is obvious she cannot go on. Plainly, she does not understand certain questions. She does not remember the use of some objects*".[6].

**Figure 3:**
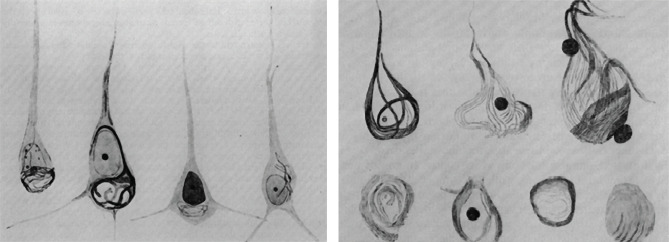
Presence of neurofibrillary tangles in the early stage (left) and terminal stage (right) of the disease [6].

After Auguste Deter's death in 1906 due to infected bedsores, Alzheimer examined her brain and noticed the presence of neuritic plaques and neurofibrillary tangles using silver staining, one of the newest techniques at that time. He concluded that there was a link between these abnormal structures and the cause of the disease (Figure 3) [7].

**Figure 4:**
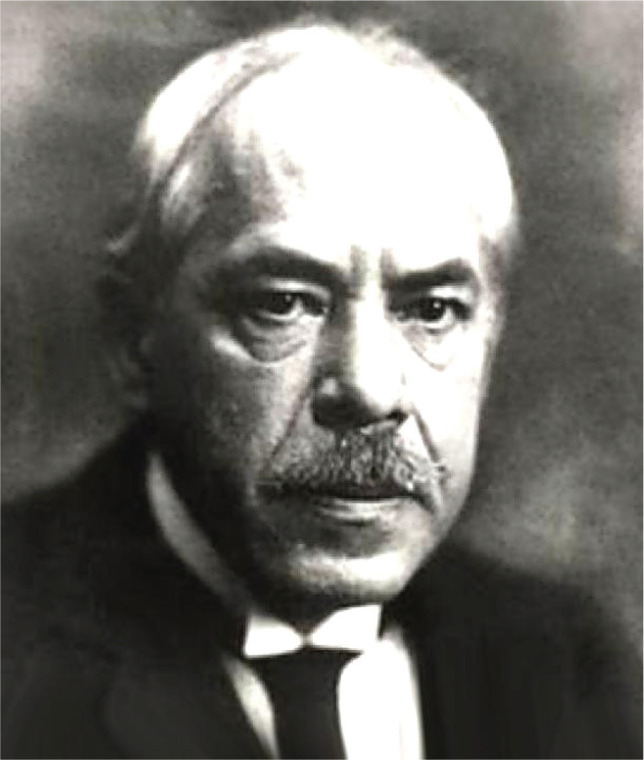
Gheorghe Marinescu (1863-1938).

In the 19^th^ century, the Romanian neurologist Gheorghe Marinescu (Figure 4), along with French pathologist Paul Blocq, discovered the existence of senile plaques (beta-amyloids) in the grey matter of eight epileptic patients' brains using hematoxylin-eosin, acidic fuchsin, and carmine staining. Later on, this discovery paved the way for Alzheimer's correlation between the discovery of senile plaques with the etiology of Alzheimer's disease [8, 9].

Neuritic plaques are the outcome of a spontaneous or genetic disorder that consists of a protein misfolding leading to aggregation of beta amyloids which comes from an amyloid precursor protein (APP) cleaved by beta and gamma-secretase.

Neurofibrillary tangles are aggregates of hyperphosphorylated tau protein that represent nowadays the most accurate paraclinical investigation that determines a clear diagnosis of Alzheimer’s disease.

Today, according to the updated Diagnostic and Statistical Manual of Mental Disorders, 5^th^ Edition (DSM-5), Alzheimer's disease can be diagnosed by fulfilling one of the following criteria: (1) the patient undergoes genetic tests that state the disease, especially if another member of the family had Alzheimer's disease or (2) all of the following three conditions are fulfilled: (a) a clear objective observation of learning or memory impairment, (b) a progressive cognitive deterioration, and (c) no evidence of other disorders such as cerebrovascular diseases, cognitive disorders, or any other neurological conditions that can assist the cognitive decline.

## THE GROWING BURDEN OF ALZHEIMER’S DISEASE

**Figure 5:**
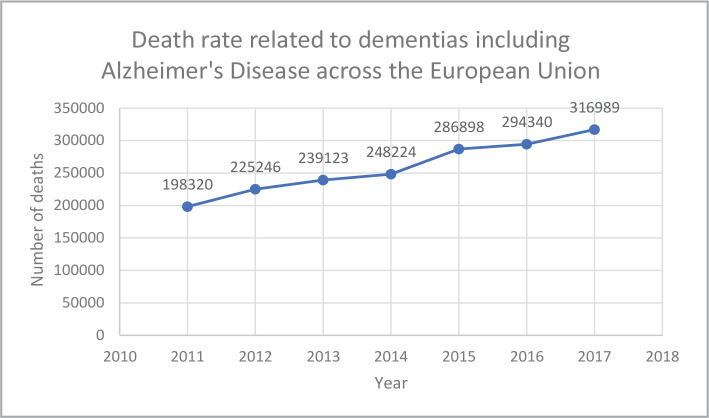
A graphic representation of the death rate among patients with dementias, including Alzheimer’s disease, across the European Union (according to Eurostat).

According to a European Union statistic, the death rate related to dementias, including Alzheimer's disease, has been on a progressively ascending path year by year, from 198,320 deaths in 2011 to 316,989 deaths in 2017, representing a 59.83% increase during these six years (Figure 5). Alzheimer's disease is by far the most common type of dementia, representing about 60-80% of all diagnosed cases of cognitive disorders [10]. In 2020, around 6.2 million Americans aged 65 or older had Alzheimer's disease, and this number is predicted to increase to 12.7 million Americans by 2050.

To comprehend this alarming statistic, we need to understand the symptoms of this pathology, the factors that can lead to Alzheimer's disease, and the currently available treatments and their benefits.

## UNDERSTANDING ALZHEIMER'S DISEASE: SYMPTOMS, RISK FACTORS, AND PREVENTION METHODS

Alzheimer's disease is a cognitive disorder that is characterized by memory loss, confusion, and difficulties completing tasks. The most prominent symptom of Alzheimer's disease is memory loss, which is caused by the presence of neurofibrillary tangles in the hippocampal neurons [11]. As the tangles spread, they change the shape of the neurons, leading to dysfunctional synapses and decreased encryption of information. Alzheimer's patients may also experience confusion when locating themselves in space and time and have difficulties completing tasks or planning events [12].

While Alzheimer's disease is easy to diagnose, there are many other factors that can lead to memory loss, including drugs, alcohol, vitamin B12 deficiency, or different brain tumors [13]. To determine whether a patient has Alzheimer's disease, a physician may ask for the family record and conduct PET/MRI scans to look for amyloids [14]. These scans can determine the presence of beta-amyloids up to 15 years before symptoms appear [15].

Alzheimer's disease is a multifactorial pathology with both genetic and environmental factors contributing to its development. However, the most significant risk factor is age, as the majority of patients with Alzheimer's are over 65 years old. According to a study conducted by Hebert L. E. *et al*. (2013) [16], the predicted prevalence for 2020 is 3.1% for people aged 65-74 years old, 16.7% for people aged 75-84 years old, and 32.2% for people aged 85 years old or more. This clearly states that an increased age determines an increased risk of developing Alzheimer’s disease.

Genetics can also play a role, but less than 1% of patients have a genetic abnormality that causes Alzheimer's disease. In those rare cases, however, a mutation to the presenilin 1 gene can almost for sure lead to Alzheimer's since this mutation will induce a protein misfolding that will produce beta-amyloid aggregation [17].

Prevention methods for Alzheimer's disease include avoiding high consumption of alcohol and cigarettes, keeping the brain active, and maintaining a healthy diet [18]. A ketogenic diet with a low carbohydrate and high fat intake can improve glucose metabolism in the brain and decrease brain amyloid-beta levels, which may prevent the occurrence of Alzheimer's disease [19, 20].

## TREATMENT OF ALZHEIMER'S DISEASE: CURRENT AVAILABLE MEDICATIONS AND FUTURE RESEARCH

Alzheimer's disease currently has a few classes of medication available for treatment. Cholinesterase inhibitors, such as Donepezil, Rivastigmine, and Galantamine, are the first possible treatment plan for patients with Alzheimer's disease. These drugs prevent the enzyme cholinesterase from destroying the acetylcholine molecules found in abnormally small quantities in the brains of patients with Alzheimer's disease. However, physicians should prescribe these drugs in the early or moderate stages of the disease [21]. In more severe cases, Memantine can be used alone or in combination with cholinesterase inhibitors to treat both cognitive and behavioral disorders, which are common symptoms in the advanced stages of the disease. Selective serotonin reuptake inhibitors (SSRIs) can also be used to treat behavioral problems and regulate mood changes in patients [5].

However, the biggest challenge in treating Alzheimer's disease is to target the beta-amyloid plaques and neurofibrillary tangles from the first occurrence to prevent the disease [22].

While there are multiple etiopathogenic explanations for this specific type of dementia, appropriate treatments for prevention have not been found yet.

Alois Alzheimer's discovery of the first patient's brain with Alzheimer's disease led to a better understanding of this new type of dementia, including its incidence, clinical manifestation, and predictive outcomes, particularly in the elderly. By sharing his findings with the scientific community, Alzheimer significantly increased knowledge about this new type of dementia and raised awareness regarding its unique clinical manifestations and prevalence. Although no appropriate treatments have been found yet, research on Alzheimer's disease is focused on finding ways to prevent the formation of beta-amyloid plaques and neurofibrillary tangles as a form of prevention.

The dedication and collaborative efforts of researchers, healthcare professionals, caregivers, and advocacy groups are critical in the fight against Alzheimer's disease, and it is our hope that continued efforts in research and treatment will lead to better outcomes for those affected by this devastating disease.
